# Author Correction: Germline de novo mutations in families with Mendelian cancer syndromes caused by defects in DNA repair

**DOI:** 10.1038/s41467-023-39587-y

**Published:** 2023-06-28

**Authors:** Kitty Sherwood, Joseph C. Ward, Ignacio Soriano, Lynn Martin, Archie Campbell, Raheleh Rahbari, Ioannis Kafetzopoulos, Duncan Sproul, Andrew Green, Julian R. Sampson, Alan Donaldson, Kai-Ren Ong, Karl Heinimann, Maartje Nielsen, Huw Thomas, Andrew Latchford, Claire Palles, Ian Tomlinson

**Affiliations:** 1 Cancer Research UK Edinburgh Centre and MRC Human Genetics Unit, Institute of Genomics and Cancer, Crewe Road, Edinburgh, EH4 2XU UK; 2 Dept of Oncology, University of Oxford, Old Road Campus Research Building, Roosevelt Drive, Oxford, OX3 7DQ UK; 3 Institute of Cancer and Genomic Sciences, University of Birmingham Medical School, Vincent Drive, Edgbaston, Birmingham, B15 2JJ UK; 4 Centre for Genetics and Experimental Medicine, Institute of Genetics and Cancer, Western General Hospital, Crewe Road, Edinburgh, EH4 2XU UK; 5 Wellcome Sanger Institute, Wellcome Genome Campus, Hinxton, UK; 6 Department of Clinical Genetics, Children’s Health Ireland and School of Medicine University College, Dublin, Ireland; 7 Institute of Medical Genetics, Division of Cancer and Genetics, Cardiff University School of Medicine, Cardiff, UK; 8 Bristol Regional Clinical Genetics Service, St Michael’s Hospital, Southwell Street, Bristol, BS2 8EG UK; 9 West Midlands Regional Genetics Service, Birmingham Women’s and Children’s NHS Foundation Trust, Birmingham, UK; 10 Institute for Medical Genetics and Pathology, University Hospital Basel, Basel, BS Switzerland; 11 Department of Clinical Genetics, Leiden University Medical Centre, 2333 ZA Leiden, the Netherlands; 12 St Mark’s Hospital, Watford Road, Harrow, HA1 3UJ UK

**Keywords:** Genetics, Cancer

Correction to: *Nature Communications* 10.1038/s41467-023-39248-0, published online 19 June 2023

The original version of this Article contained an error in Fig. 1, in which panel d was inadvertently a duplicate of panel a. This has now been corrected both in the html and pdf version.

