# Supplementary material for: Germline de novo mutations in families with Mendelian cancer syndromes caused by defects in DNA repair
Source: Nat Commun. 2023 Jun 19;14:3636. doi: 10.1038/s41467-023-39248-0 (PMC10279637; doi:10.1038/s41467-023-39248-0)
Supplement: Supplementary file 1 — Supplementary Information [file 41467_2023_39248_MOESM1_ESM.pdf]

SUPPLEMENTARY INFORMATION

Supplementary Figure 1. Pedigrees of the families studied. a) Families with parental *POLE* p.L424V germline mutation. b) Families with parental *POLD1* p.S478N germline mutation. c) Families with parental *MUTYH* germline mutations, with heterozygote and homozygote carriers distinguisgable by the specific protein changes shown. d) Control families with no known mutations in DNA repair or other Mendelian disease genes. Filled symbol = affected with phenotype shown. + = germline mutation carrier, - = non-carrier. Cumulative numbers of colorectal adenomas, polyps and hyperplastic polyps (HPs) to date of study recruitment and age at that time are given where known. For cancers, the age provided is at first presentation.

*POLE*_B

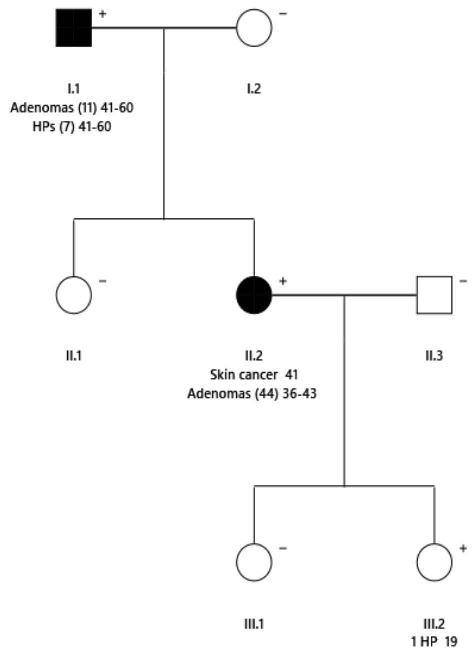

a) *POLE*_A

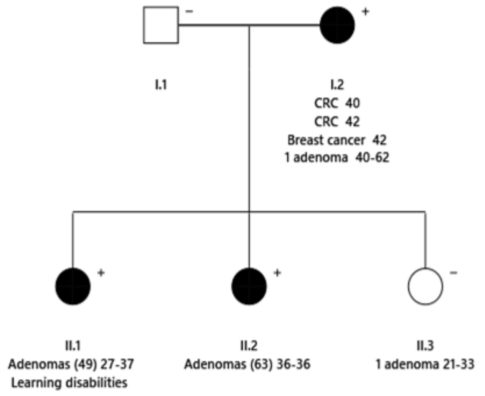

b) *POLD1*_A

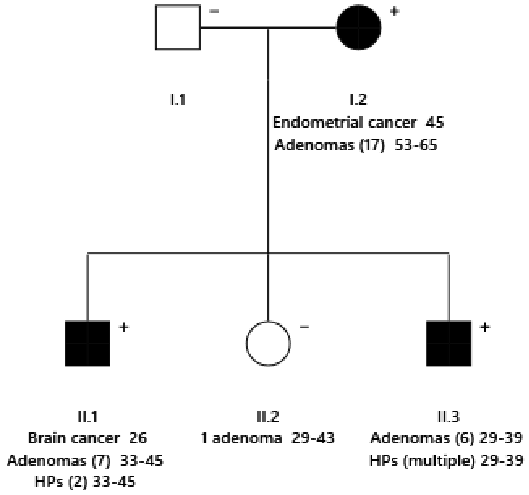

+

*POLD1*_B

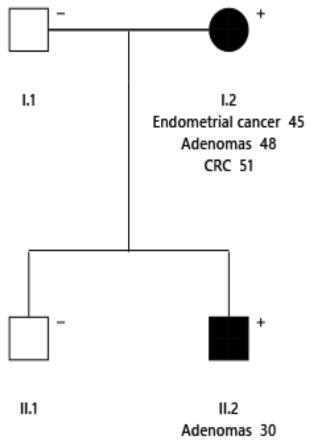

c)

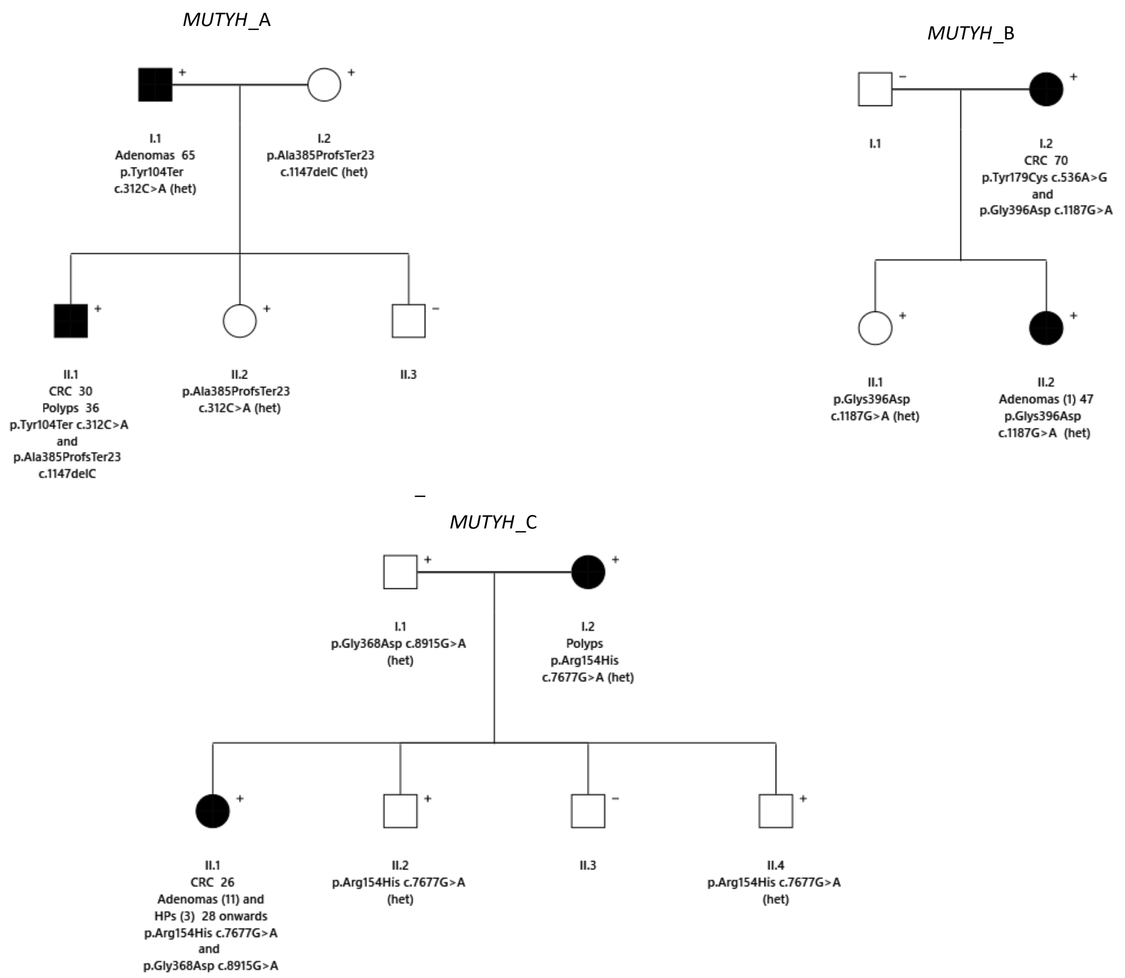
MUTYH_A
I.1
Adenomas 65
p.Tyr104Ter
c.312C>A (het)
I.2
p.Ala385ProfsTer23
c.1147delC (het)
II.1
CRC 30
Polyps 36
p.Tyr104Ter c.312C>A
and
p.Ala385ProfsTer23
c.1147delC
II.2
p.Ala385ProfsTer23
c.312C>A (het)
II.3
MUTYH_B
I.1
I.2
CRC 70
p.Tyr179Cys c.536A>G
and
p.Gly396Asp c.1187G>A
II.1
p.Glys396Asp
c.1187G>A (het)
II.2
Adenomas (1) 47
p.Glys396Asp
c.1187G>A (het)
MUTYH_C
I.1
p.Gly368Asp c.8915G>A
(het)
I.2
Polyps
p.Arg154His
c.7677G>A (het)
II.1
CRC 26
Adenomas (11) and
HPs (3) 28 onwards
p.Arg154His c.7677G>A
and
p.Gly368Asp c.8915G>A
II.2
p.Arg154His c.7677G>A
(het)
II.3
II.4
p.Arg154His c.7677G>A
(het)

d)

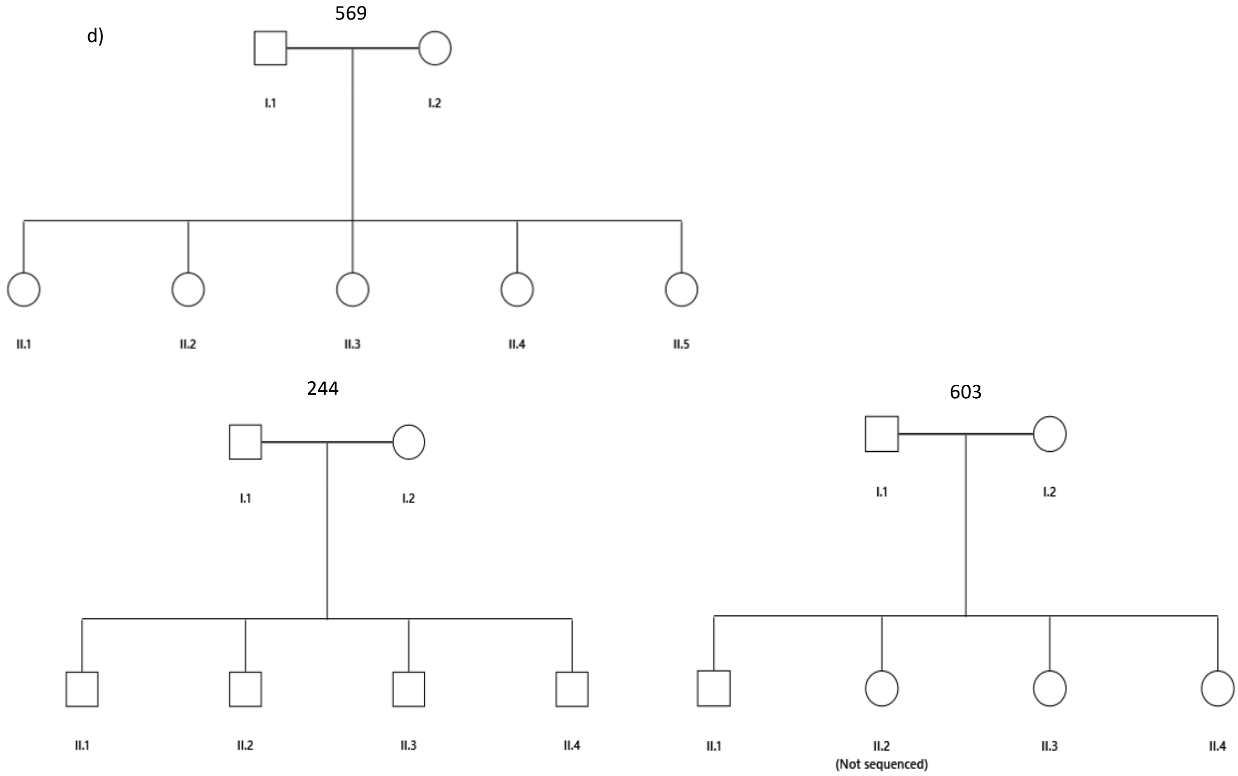
569
I.1
I.2
II.1
II.2
II.3
II.4
II.5
244
I.1
I.2
II.1
II.2
II.3
II.4
603
I.1
I.2
II.1
II.2
(Not sequenced)
II.3
II.4

c)

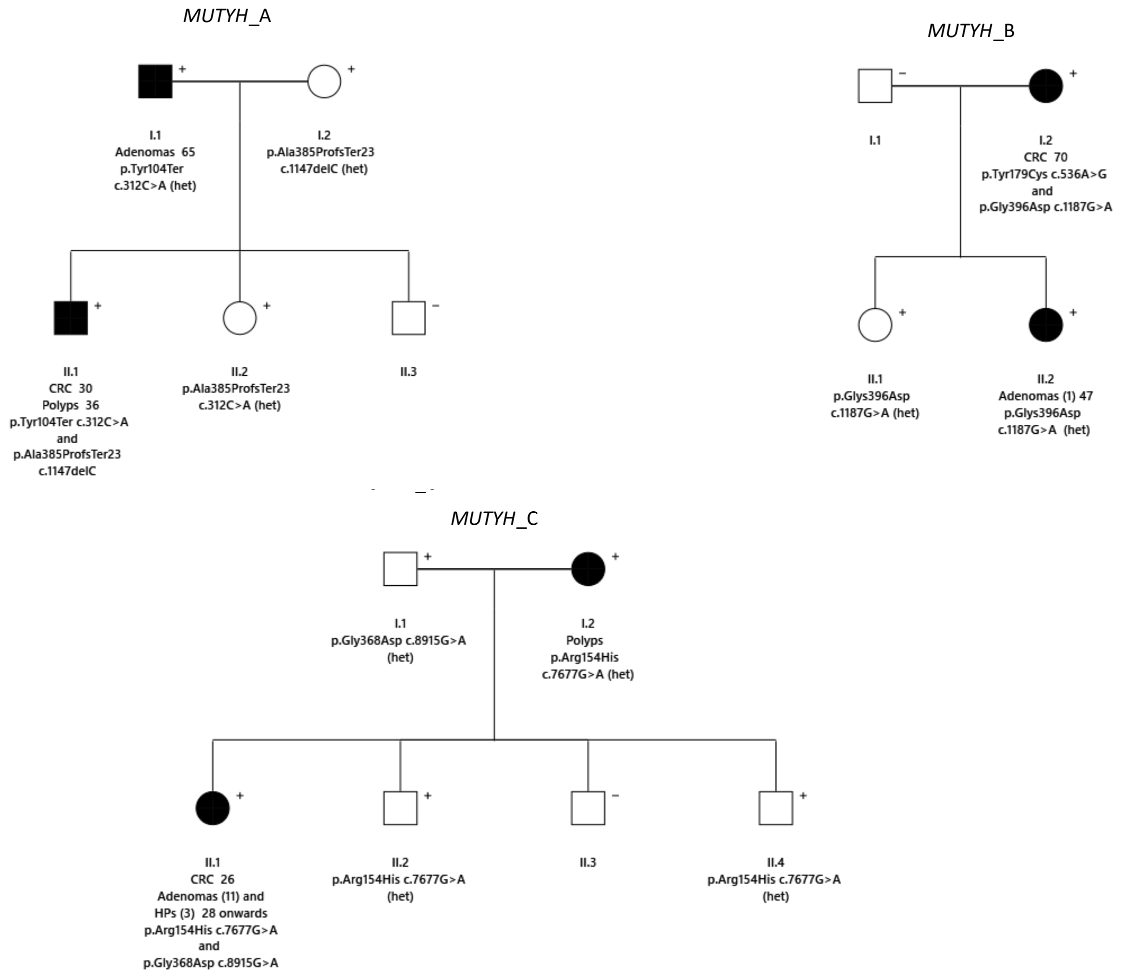

*MUTYH*_A
+
+
I.1
Adenomas 65
p.Tyr104Ter
c.312C>A (het)
I.2
p.Ala385ProfsTer23
c.1147delC (het)
+
+
−
II.1
CRC 30
Polyps 36
p.Tyr104Ter c.312C>A
and
p.Ala385ProfsTer23
c.1147delC
II.2
p.Ala385ProfsTer23
c.312C>A (het)
II.3
*MUTYH*_B
−
+
I.1
I.2
CRC 70
p.Tyr179Cys c.536A>G
and
p.Gly396Asp c.1187G>A
+
+
II.1
p.Glys396Asp
c.1187G>A (het)
II.2
Adenomas (1) 47
p.Glys396Asp
c.1187G>A (het)
*MUTYH*_C
+
+
I.1
p.Gly368Asp c.8915G>A
(het)
I.2
Polyps
p.Arg154His
c.7677G>A (het)
+
+
−
+
II.1
CRC 26
Adenomas (11) and
HPs (3) 28 onwards
p.Arg154His c.7677G>A
and
p.Gly368Asp c.8915G>A
II.2
p.Arg154His c.7677G>A
(het)
II.3
II.4
p.Arg154His c.7677G>A
(het)

d)

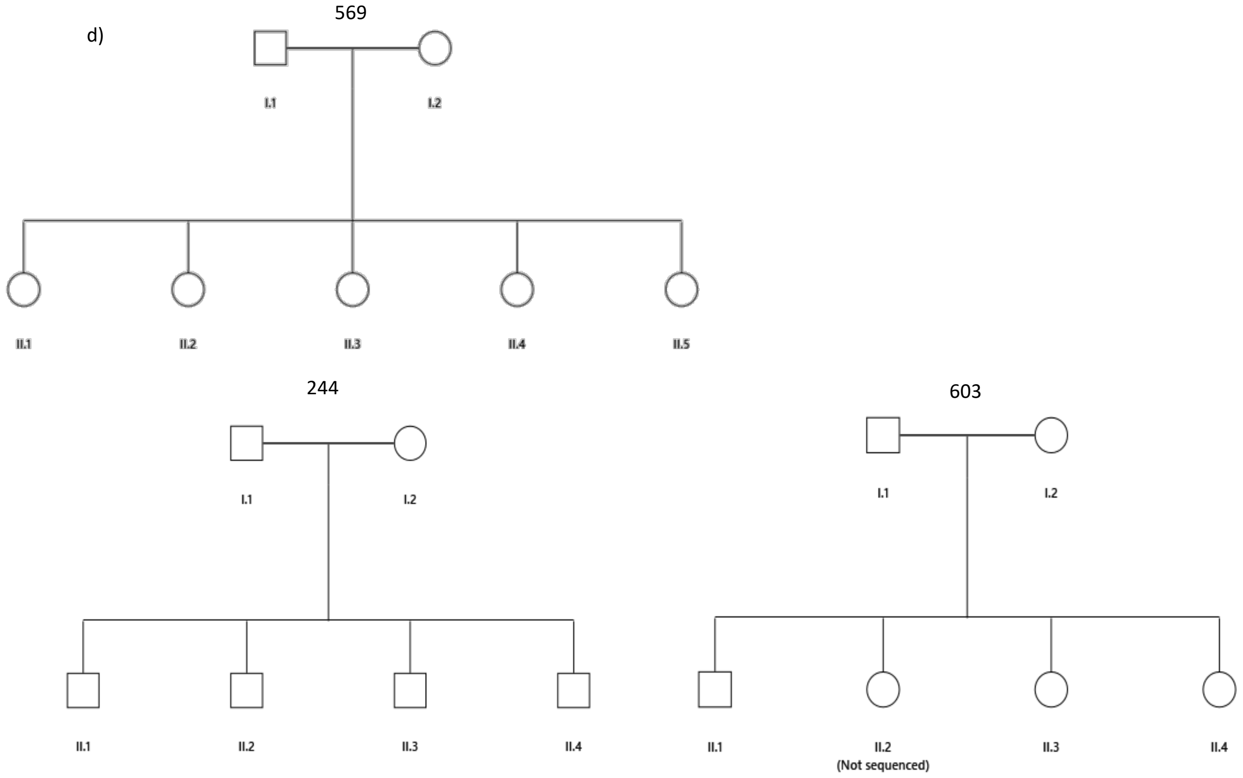

569
I.1
I.2
II.1
II.2
II.3
II.4
II.5
244
I.1
I.2
II.1
II.2
II.3
II.4
603
I.1
I.2
II.1
II.2
(Not sequenced)
II.3
II.4

Supplementary Figure 2. DNM assessment. a) Workflow for filtering de novo mutations in parent-offspring trios from DeNovoGear. b) An example of a candidate de novo mutation that is validated in the IGV browser. The read alignments from top to bottom are the child's, mother's and father's. The DNM is present only in the child's reads.

a)

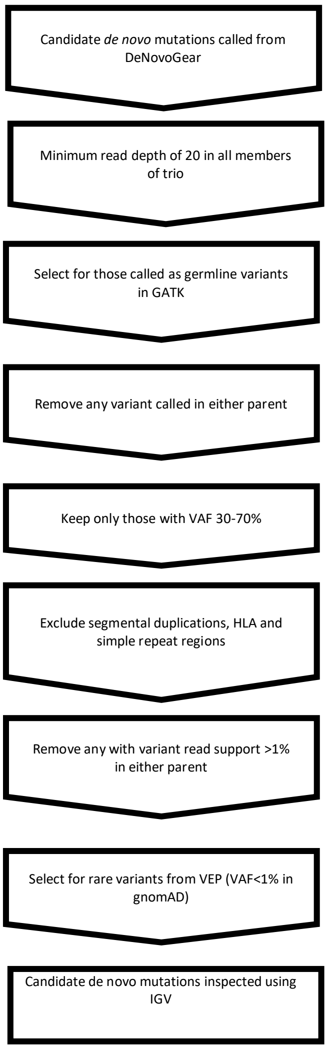

b)

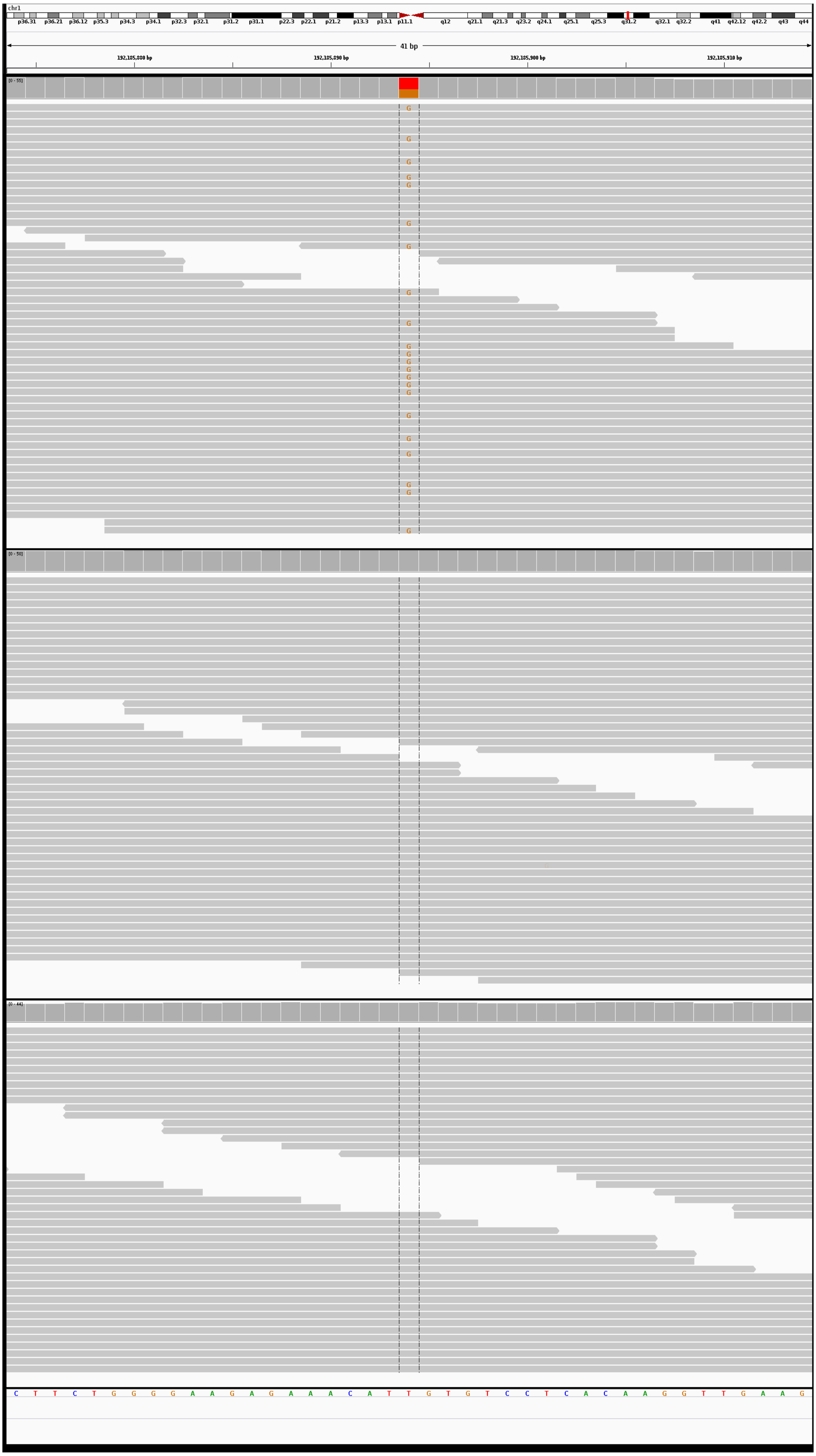
chr1
41 bp
192,105,880 bp
192,105,890 bp
192,105,900 bp
192,105,910 bp
C T T C T G G G G A A G A G A A A C A T T G T G T C C T C A C A A G G T T G A A G

Supplementary Figure 3. Exploration of different reference genomes. We had used reference genome hg19/B37 (with patches) in large part to achieve comparability with previous studies. Given the improved coverage of hg38/B38, we re-mapped and called three individuals from family POLE_A using B38. The results are shown below for SBS DNMs (B37 blue, B38 red).

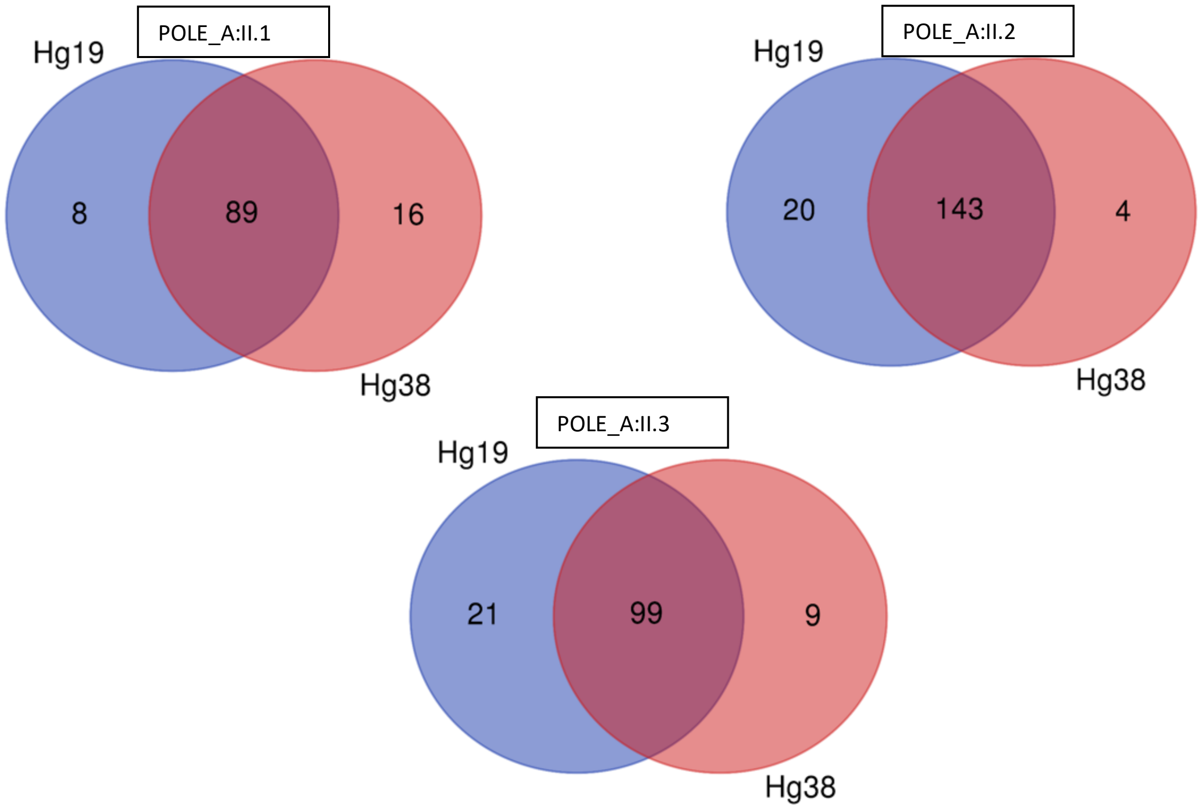

On average, the number of DNMs identified by B38 was similar to B37 (within 10% either way), with 80% overlap of specific DNMs. There was no clear DNM increase in the B38 analysis in this small sample. Visual inspection to check and assess the DNMs suggested that the differences between DNM identification between reference builds resulted from quality score differences, presumably reflecting the interplay between our sequencing errors and errors in the reference sequences.

Supplementary Figure 4. Activities of SBS, DBS and ID mutation signatures of all families. a) All POLE families; b) POLE_A; c) POLE_B all; d) POLE_B generation II; e) POLE_B generation III; f) All POLD1 families; g) POLD1_A; h) POLD1_B; i) All MUTYH families; j) MUTYH_A; k) MUTYH_B; l) MUTYH_C; m) All Control families; n) Control 244; o) Control 569; p) Control 603.

a)

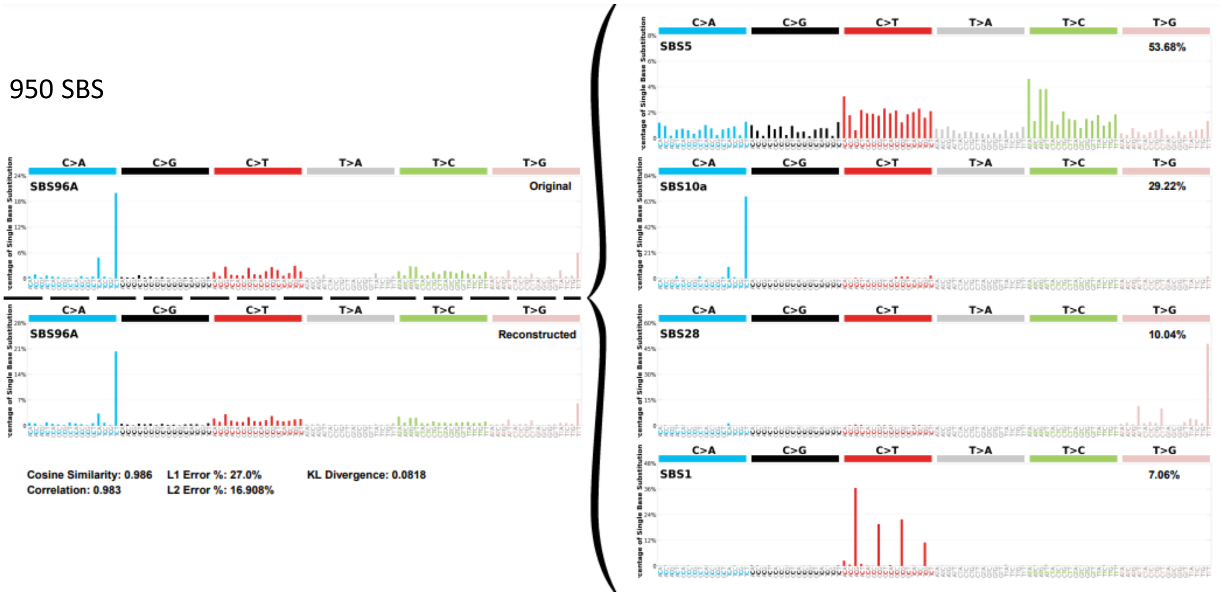

b)

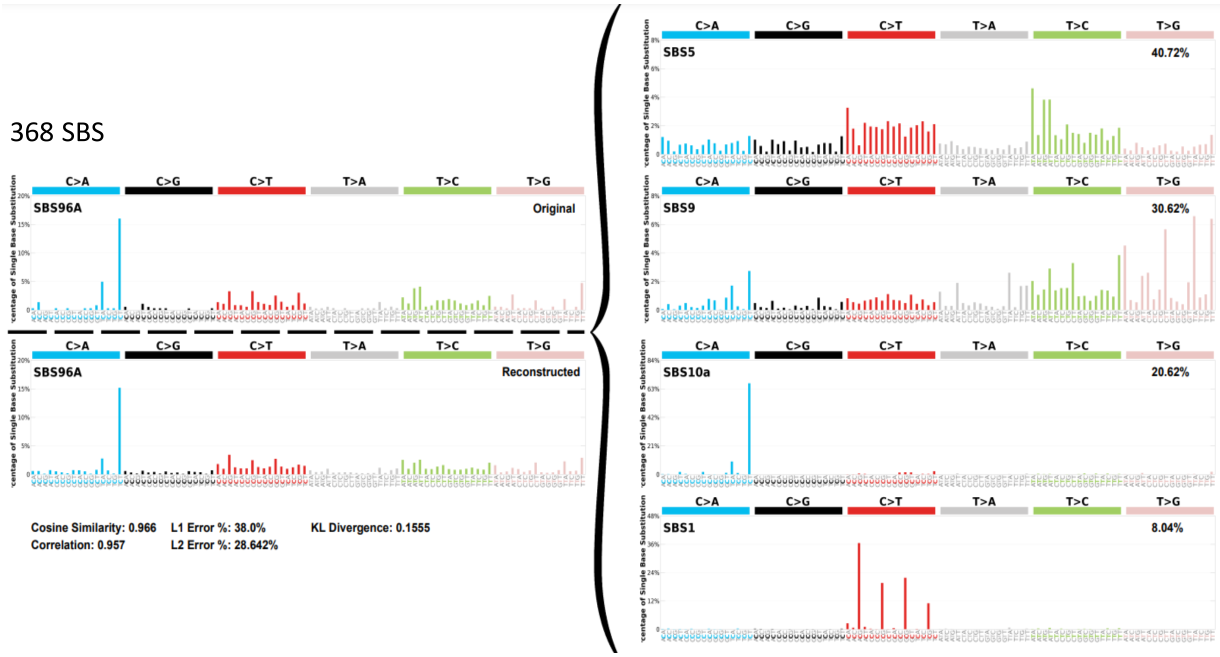

c)

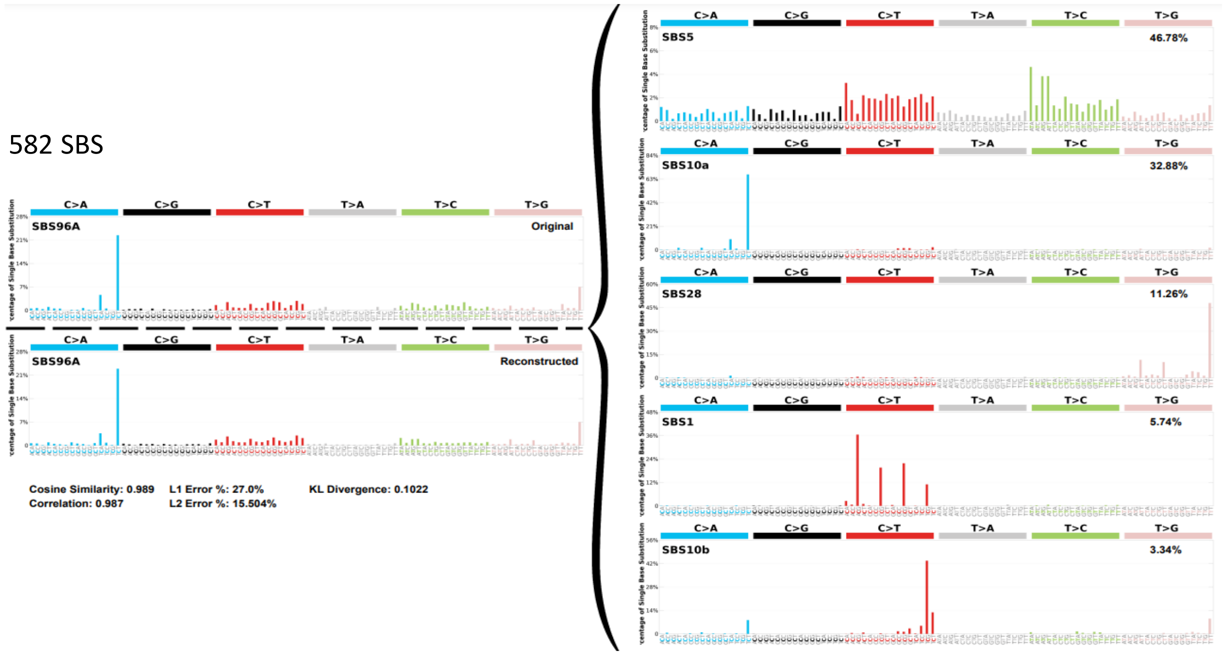
582 SBS
SBS96A Original
SBS96A Reconstructed
Cosine Similarity: 0.989
Correlation: 0.987
L1 Error %: 27.0%
L2 Error %: 15.504%
KL Divergence: 0.1022
C>A C>G C>T T>A T>C T>G
SBS5 46.78%
SBS10a 32.88%
SBS28 11.26%
SBS1 5.74%
SBS10b 3.34%

d)

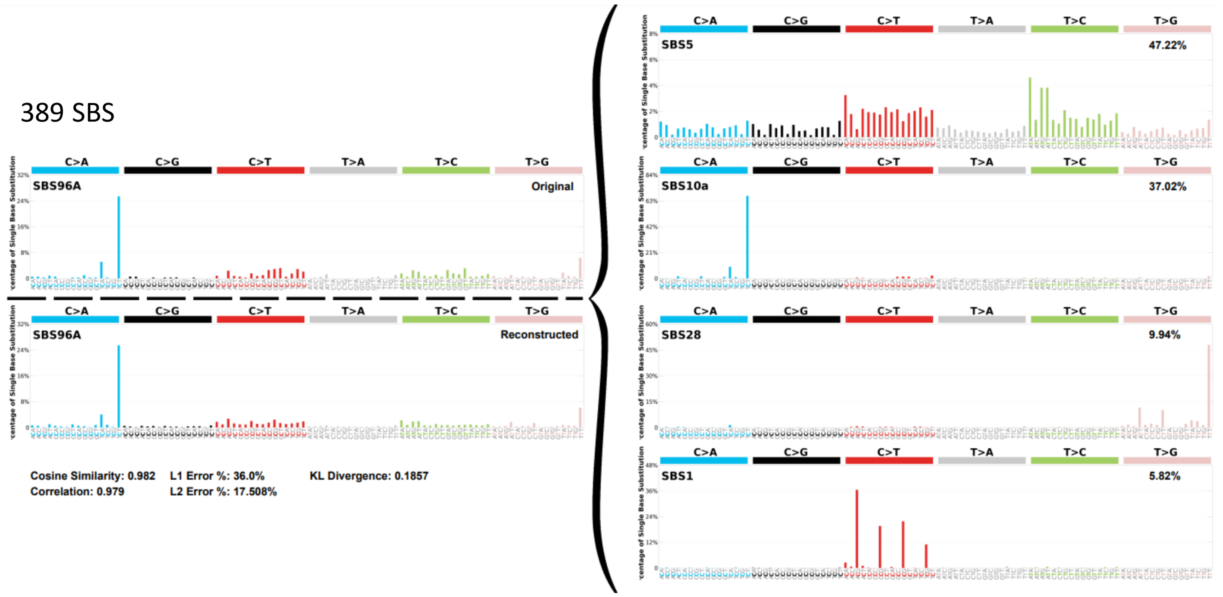
389 SBS
SBS96A Original
SBS96A Reconstructed
Cosine Similarity: 0.982
Correlation: 0.979
L1 Error %: 36.0%
L2 Error %: 17.508%
KL Divergence: 0.1857
C>A C>G C>T T>A T>C T>G
SBS5 47.22%
SBS10a 37.02%
SBS28 9.94%
SBS1 5.82%

e)

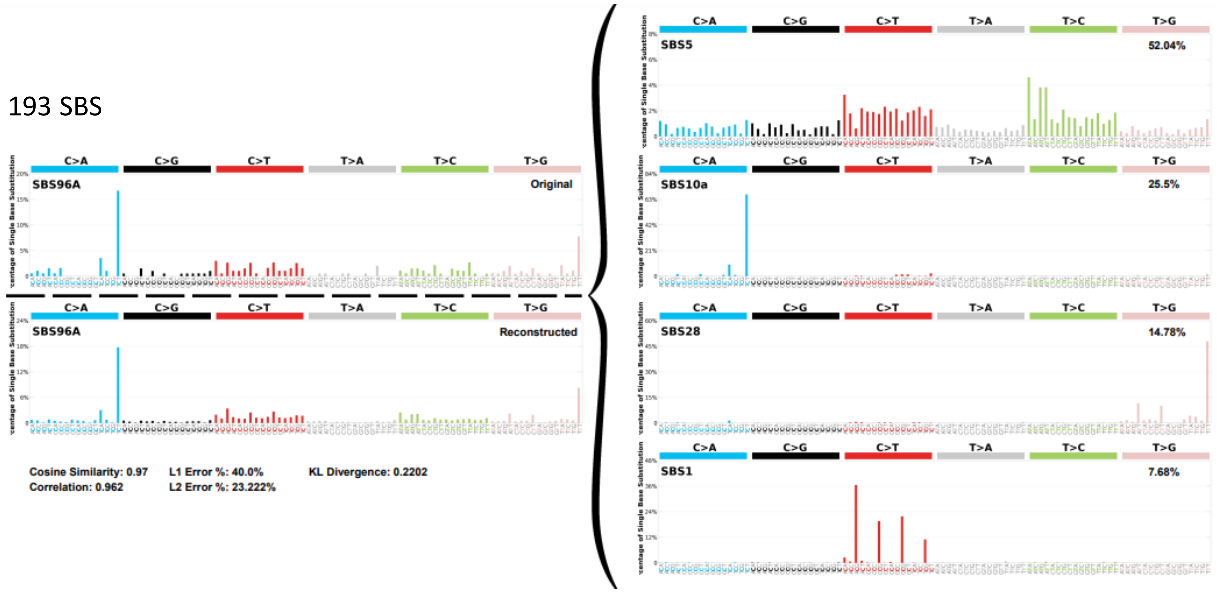
193 SBS
C>A
C>G
C>T
T>A
T>C
T>G
SBS96A
Original
SBS96A
Reconstructed
Cosine Similarity: 0.97
Correlation: 0.962
L1 Error %: 40.0%
L2 Error %: 23.222%
KL Divergence: 0.2202
SBS5
52.04%
SBS10a
25.5%
SBS28
14.78%
SBS1
7.68%

f)

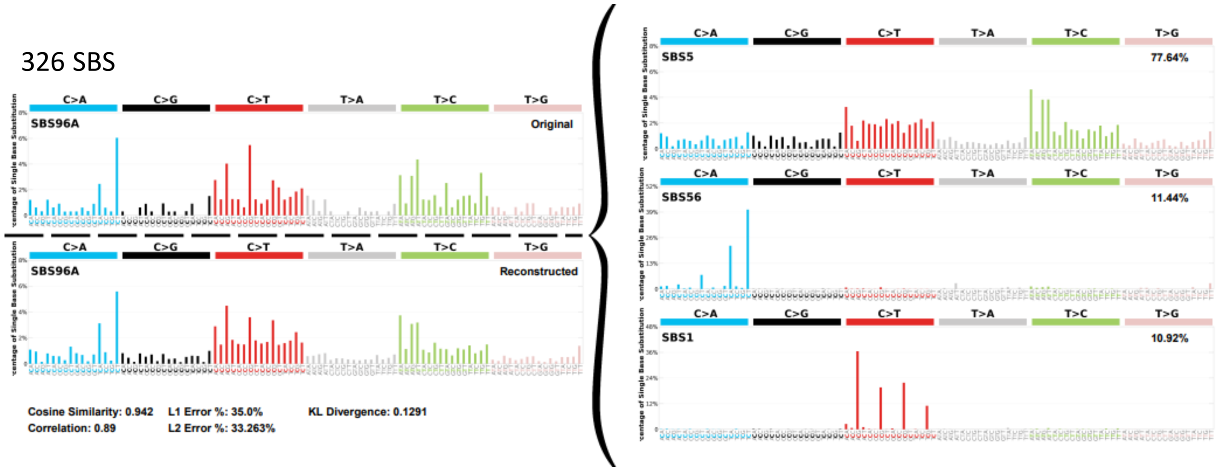
326 SBS
C>A
C>G
C>T
T>A
T>C
T>G
SBS96A
Original
SBS96A
Reconstructed
Cosine Similarity: 0.942
Correlation: 0.89
L1 Error %: 35.0%
L2 Error %: 33.263%
KL Divergence: 0.1291
SBS5
77.64%
SBS56
11.44%
SBS1
10.92%

g)

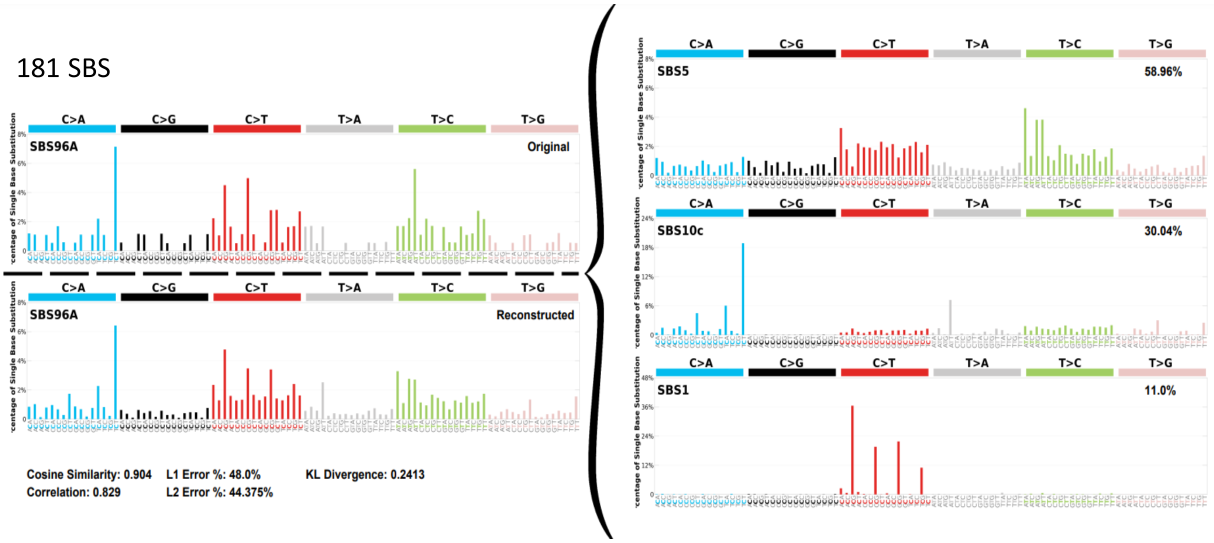
181 SBS
C>A
C>G
C>T
T>A
T>C
T>G
SBS96A
Original
SBS96A
Reconstructed
Cosine Similarity: 0.904
Correlation: 0.829
L1 Error %: 48.0%
L2 Error %: 44.375%
KL Divergence: 0.2413
SBS5
58.96%
SBS10c
30.04%
SBS1
11.0%

h)

145 SBS

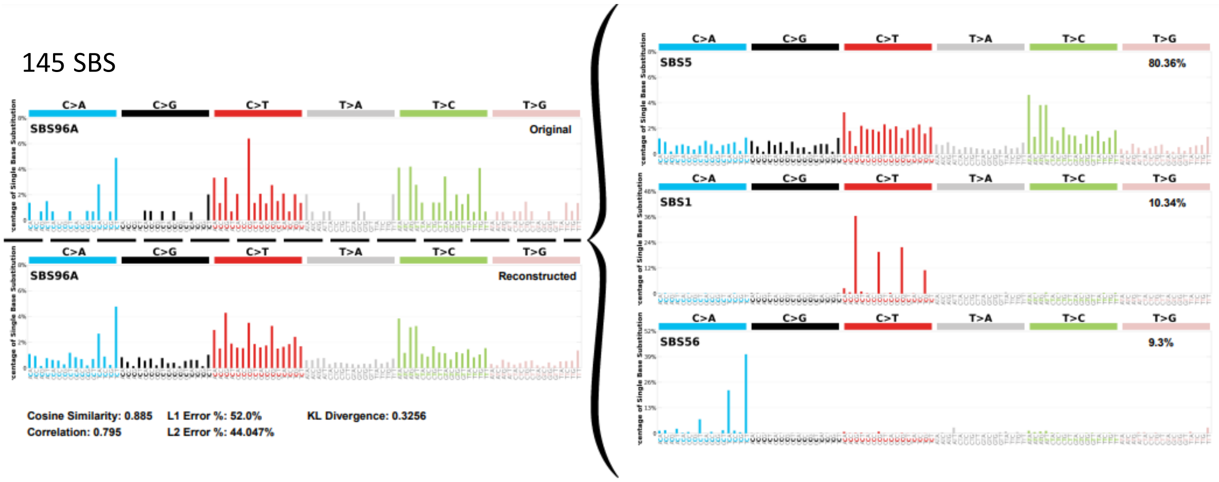

i)

475 SBS

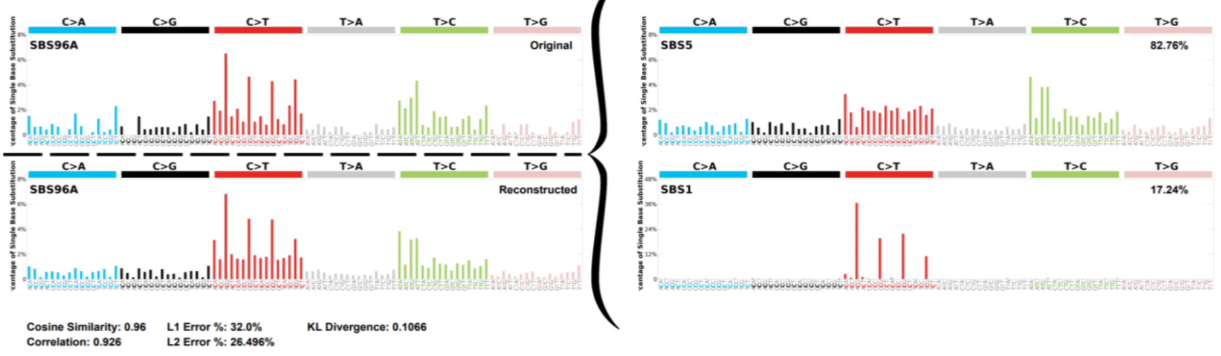

j)

166 SBS

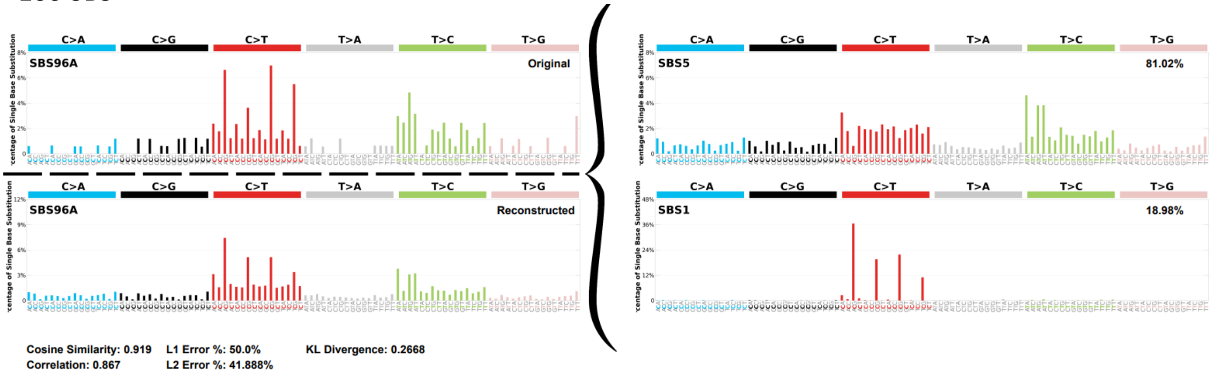

k)

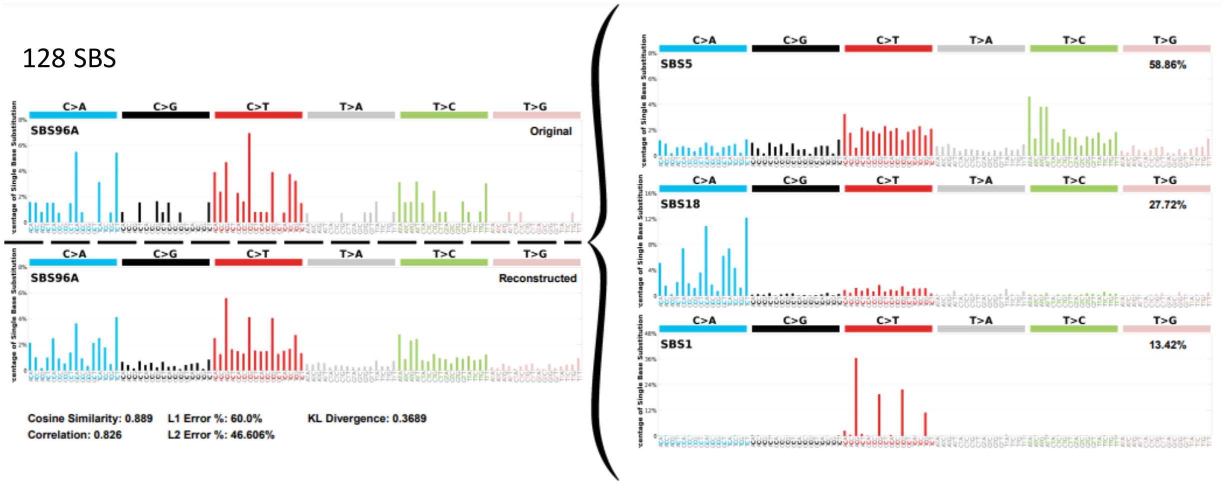

128 SBS
C>A
C>G
C>T
T>A
T>C
T>G
SBS96A
Original
SBS96A
Reconstructed
Cosine Similarity: 0.889
Correlation: 0.826
L1 Error %: 60.0%
L2 Error %: 46.606%
KL Divergence: 0.3689
SBS5
58.86%
SBS18
27.72%
SBS1
13.42%

l)

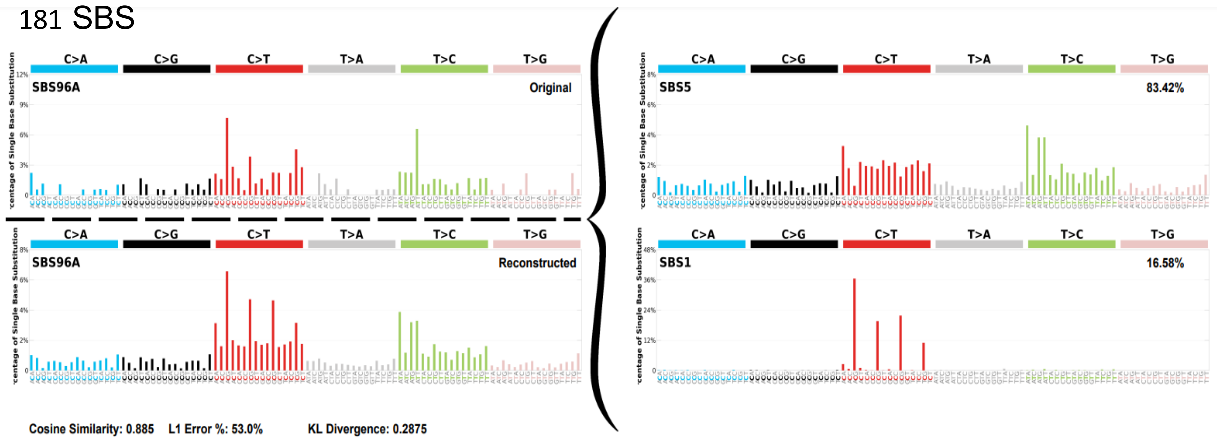

181 SBS
C>A
C>G
C>T
T>A
T>C
T>G
SBS96A
Original
SBS96A
Reconstructed
Cosine Similarity: 0.885
Correlation: 0.8
L1 Error %: 53.0%
L2 Error %: 49.46%
KL Divergence: 0.2875
SBS5
83.42%
SBS1
16.58%

m)

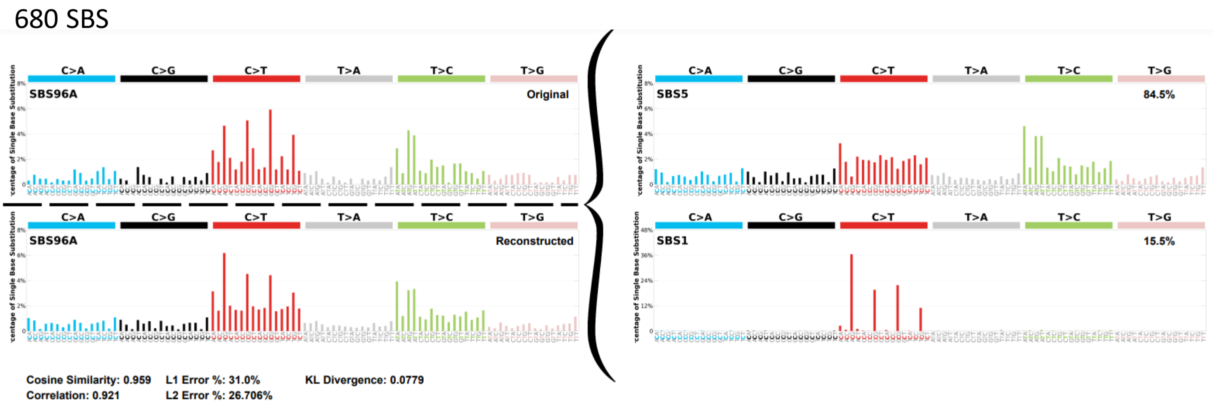

680 SBS
C>A
C>G
C>T
T>A
T>C
T>G
SBS96A
Original
SBS96A
Reconstructed
Cosine Similarity: 0.959
Correlation: 0.921
L1 Error %: 31.0%
L2 Error %: 26.706%
KL Divergence: 0.0779
SBS5
84.5%
SBS1
15.5%

n)

219 SBS

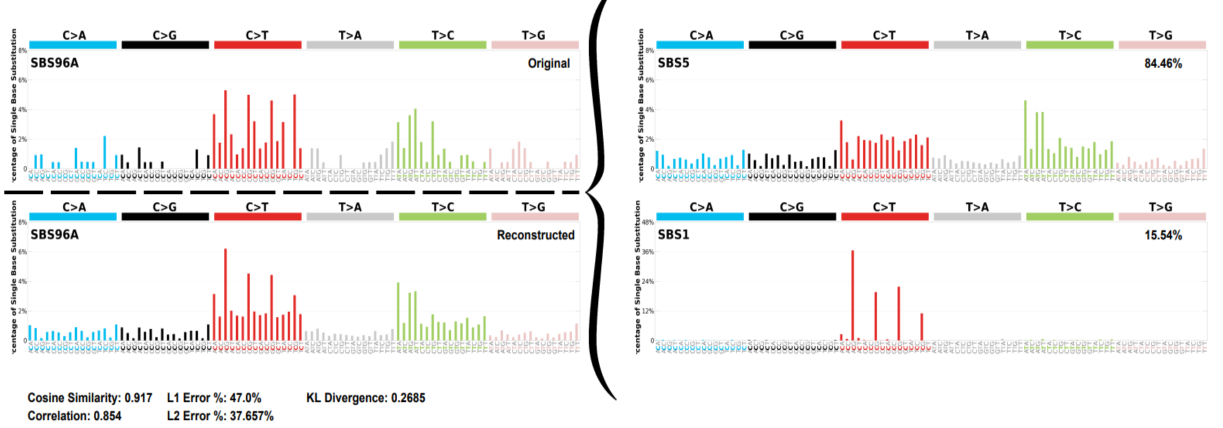

o)

281 SBS

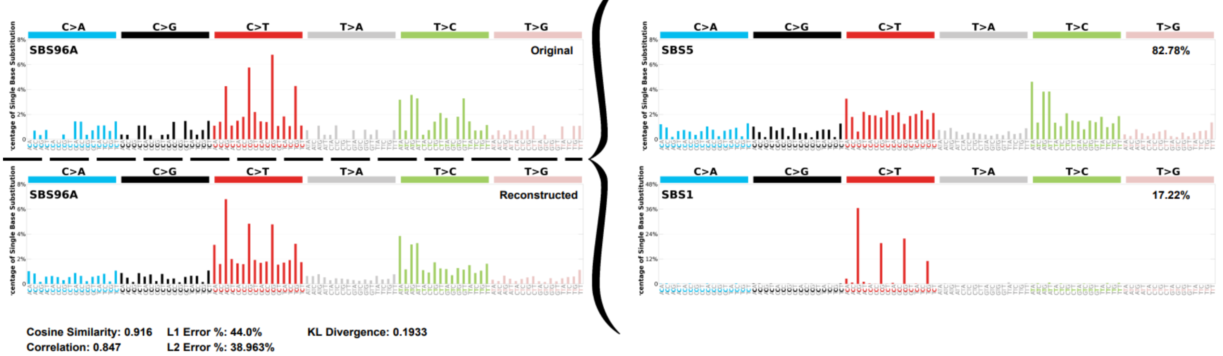

p)

180 SBS

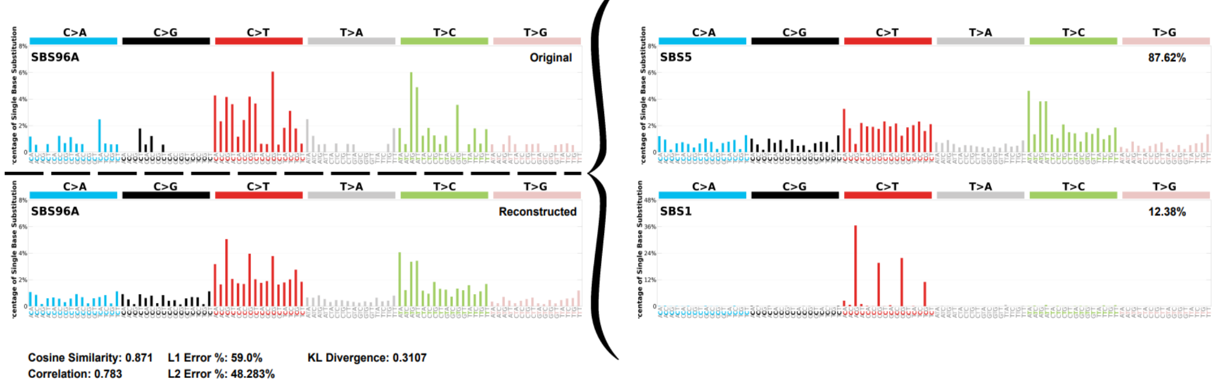

Supplementary Figure 5. Proportional activities of SBS signatures derived from mutations phased to carrier and non-carrier parents in *POLE* and *POLD1* families. a) POLE carrier parents, b) POLE non-carrier parents, c) POLD1 carrier parents, d) POLD1 non-carrier parents. Note the presence of signature 10d in the mutations derived from the POLE non-carrier parents. These mutations do not have a clear cause, but were almost all derived from a father who provided DNMs assigned to SBS56, which is formally assigned as an artefact by COSMIC, but closely resembles SBS10d.

a)

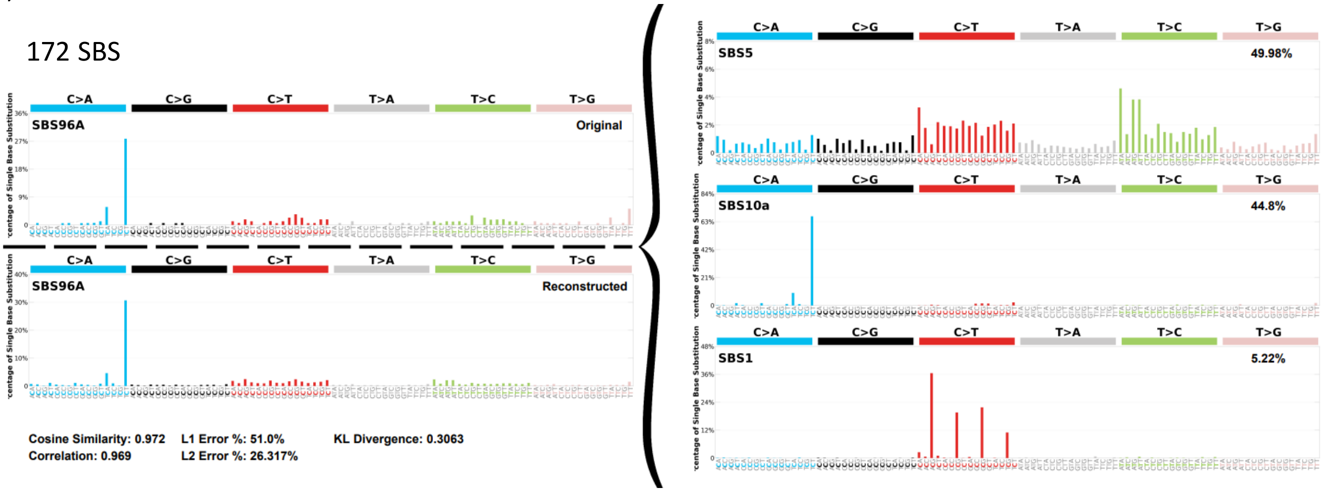

b)

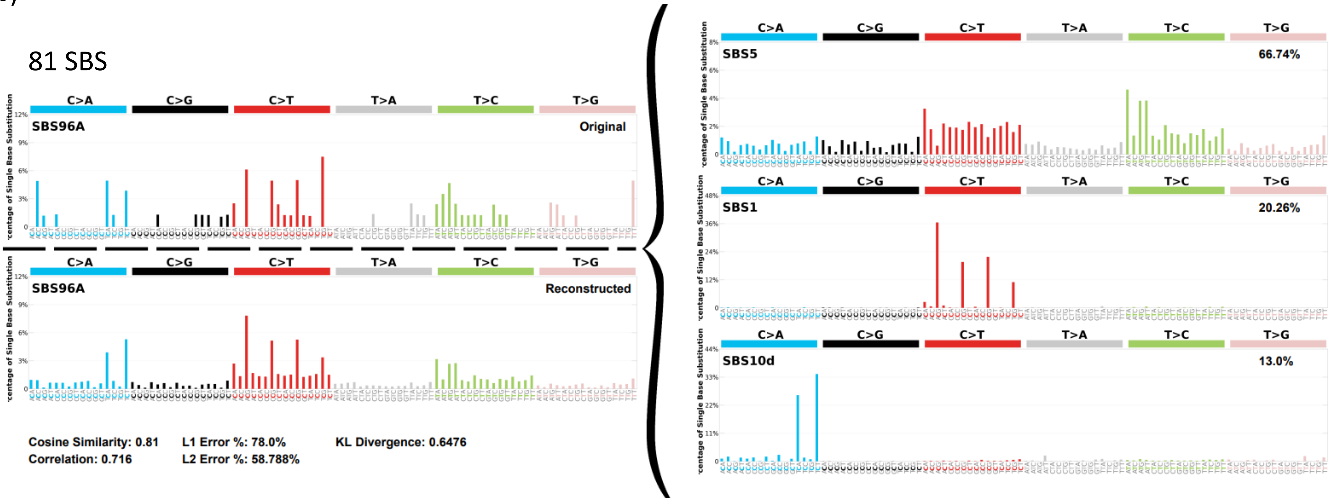

c)

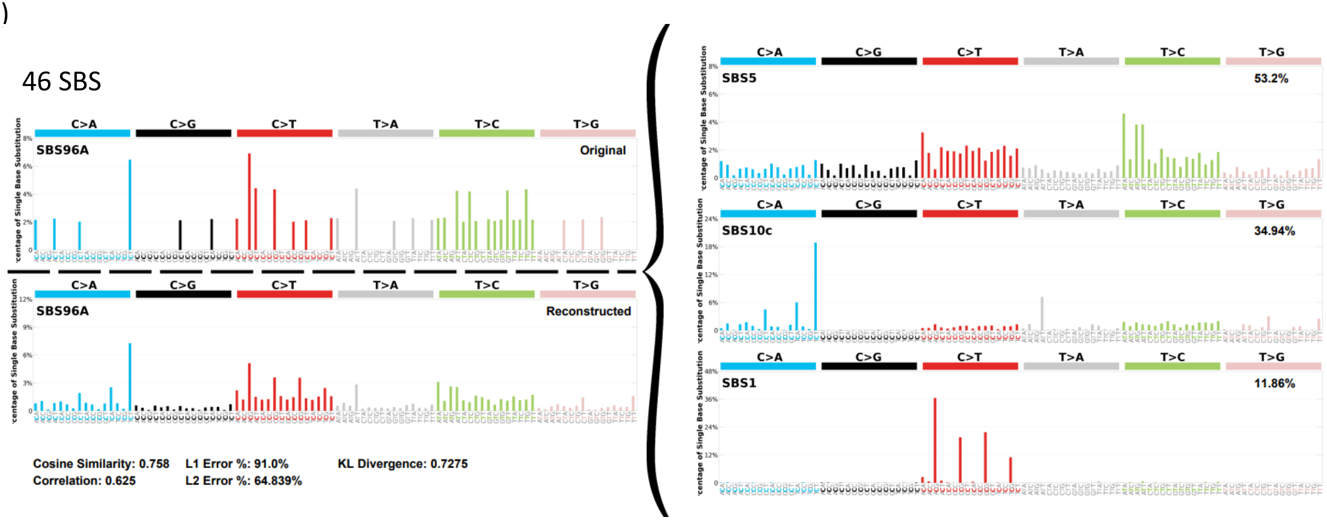

46 SBS
C>A
C>G
C>T
T>A
T>C
T>G
SBS96A
Original
Reconstructed
Cosine Similarity: 0.758
Correlation: 0.625
L1 Error %: 91.0%
L2 Error %: 64.839%
KL Divergence: 0.7275
SBS5
53.2%
SBS10c
34.94%
SBS1
11.86%

d)

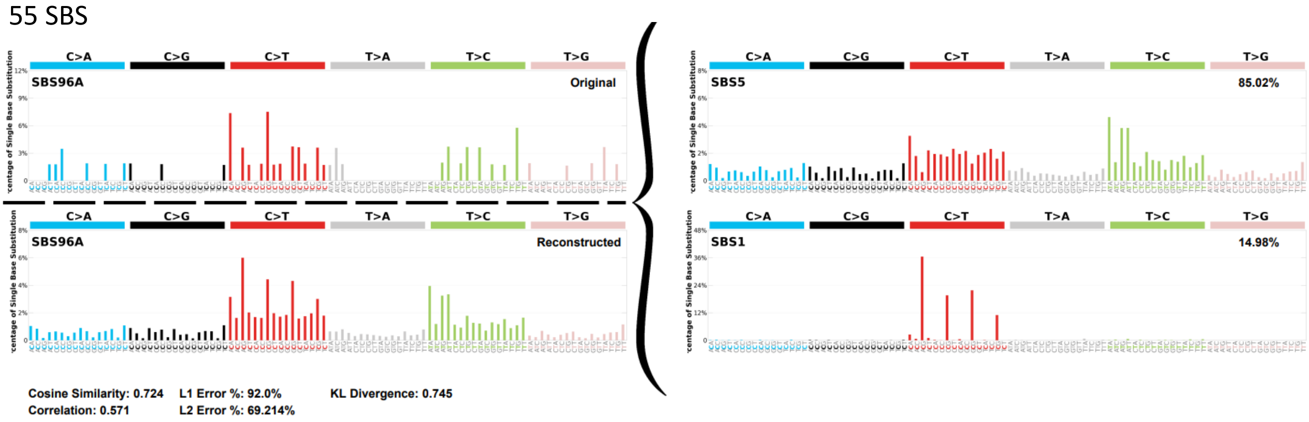

55 SBS
C>A
C>G
C>T
T>A
T>C
T>G
SBS96A
Original
Reconstructed
Cosine Similarity: 0.724
Correlation: 0.571
L1 Error %: 92.0%
L2 Error %: 69.214%
KL Divergence: 0.745
SBS5
85.02%
SBS1
14.98%

Supplementary Figure 6. VAF distributions of DNMs of all children. Shapiro-Wilk test for normality statistic and P value are shown. Note the deviations from a symmetrical distribution centered on VAF=0.5 in a few cases, generally consistent with left skewing owing to detection of a small number of sub-clonal post-zygotic mutations. Nevertheless, MUTYH_C:II.1 remains a clear outlier in terms of both VAF distribution and deviation from normality.

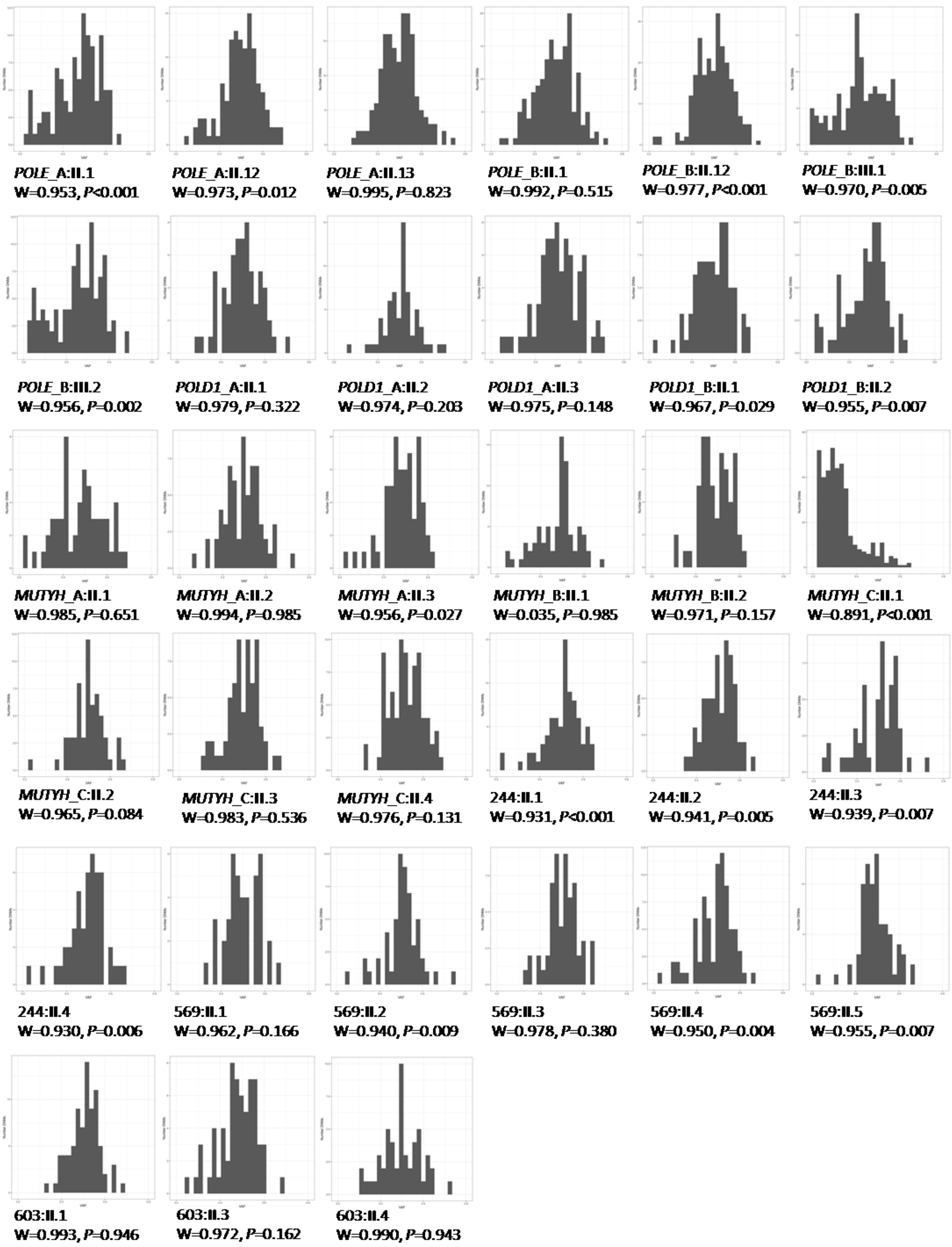

Supplementary Figure 7. *MUTYH*_C:II.1 analysis.

a) DNM frequency by VAF. For this patient only, we performed an additional analysis in which lower confidence DNMs at lower VAFs were included, in order to investigate the hypothesis that there existed a sub-clonal peak of changes related to oligoclonal haematopoiesis after chemotherapy. Gaussian mixture analysis identified two groups, which we found to correspond to the peaks at VAF~0.23 and VAF~0.48, as shown red and blue in the histogram, with a separation at approximately VAF=0.36-0.40. We surmise that these groups corresponded respectively to a large sub-clonal peak of therapy-associated mutations and a clonal peak of true DNMs. Note that full visual inspection of every DNM was not performed for this large set of 960 mutations, so burdens are not equivalent to the high-confidence set of DNMs shown for all study patients in Table 1.

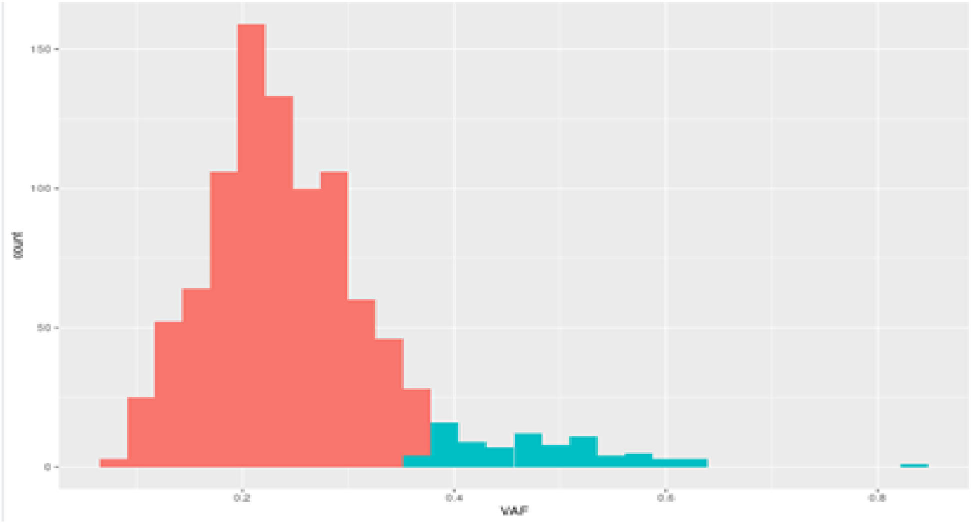

b) Cosine similarities of DNMs from all children in the study to various reported 5- fluorouracil signatures. There is no enrichment of these signatures in MUTYH_C:II.1. Signatures are derived from COSMIC (https://cancer.sanger.ac.uk/signatures/) or from {Pich, 2021 #42}.

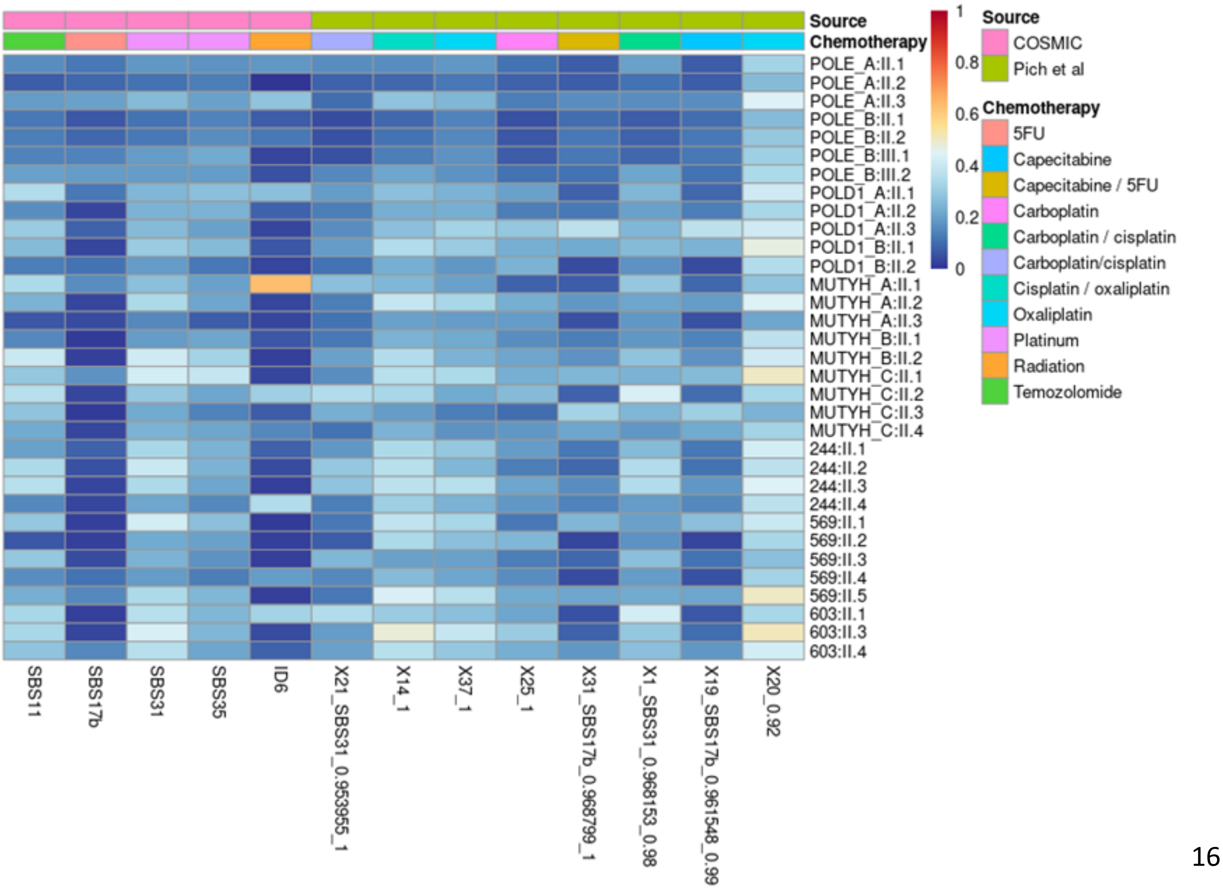

Supplementary Table 1. Number of variants remaining at each stage of calling and filtering.

Child ID	GATK germline variants	Raw DeNovo Gear	Called as variant in GATK	Not called as variant in parents	VAF 0.3-0.7	<1% read support in both parents	Exclude simple repeats	Exclude segmental duplications	Remove HLA region	VAF<0.01 in gnomAD	Validated in IGV
POLE_A:II.1	2531366	1756	1292	1264	442	404	226	180	180	179	97
POLE_A:II.2	2542600	1518	1108	1107	368	337	186	149	149	149	120
POLE_A:II.3	2533887	1659	1038	1037	396	363	212	188	188	188	163
POLE_B:II.1	2464024	1401	1065	1065	321	299	207	192	192	192	161
POLE_B:II.2	2485269	1390	938	938	400	375	275	256	256	255	239
POLE_B:III.1	2495072	1468	942	941	301	278	165	150	150	150	114
POLE_B:III.2	2489240	1439	1047	1041	296	270	146	123	123	123	88
POLD1_A:II.1	2532151	1334	1004	1004	235	222	122	103	103	103	63
POLD1_A:II.2	2510682	1120	819	819	248	228	92	78	78	78	62
POLD1_A:II.3	2512732	1110	843	843	251	238	110	97	97	97	71
POLD1_B:II.1	2514464	1484	1180	1180	300	276	149	110	110	110	83
POLD1_B:II.2	2520854	1813	1239	1239	331	311	146	138	138	138	76
MUTYH_A:II.1	2565987	2125	1874	1857	307	285	148	101	101	101	57
MUTYH_A:II.2	2560630	1527	977	973	275	259	129	94	94	94	63
MUTYH_A:II.3	2563090	1319	944	944	244	230	118	89	89	89	59
MUTYH_B:II.1	2534270	1560	1258	1258	268	244	143	121	121	121	76
MUTYH_B:II.2	2515758	1175	952	952	232	213	109	102	102	102	60
MUTYH_C:II.1	2491493	2077	1744	1716	537	513	315	288	288	288	212
MUTYH_C:II.2	2523298	1374	868	868	299	269	122	104	104	104	59
MUTYH_C:II.3	2506464	1163	644	643	244	230	121	94	94	94	64
MUTYH_C:II.4	2514724	1165	856	855	313	290	139	118	118	118	81
244:II.1	2518039	1134	791	790	299	281	159	133	133	133	79
244:II.2	2515097	1125	823	822	250	230	105	81	81	81	62
244:II.3	2505711	1270	895	893	260	231	108	83	83	83	50
244:II.4	2506701	1452	1118	1091	267	243	105	79	79	79	47
569:II.1	2508402	1124	662	659	211	195	78	63	63	63	42
569:II.2	2505892	1111	696	693	218	199	85	67	67	67	53
569:II.3	2519274	1194	668	667	208	186	89	73	72	72	58
569:II.4	2497746	1419	709	706	232	220	105	88	88	88	75
569:II.5	2499785	1445	829	807	307	286	125	104	104	104	78
603:II.1	2513538	1789	1211	1208	295	261	130	108	108	108	80
603:II.3	2535778	1635	1155	1122	323	290	115	92	92	92	62
603:II.4	2510779	1344	1078	1076	260	240	86	72	72	72	51

Supplementary Table 2. Six-channel DNM spectra in *POLE, POLD1, MUTYH* and control families. Total numbers of DNMs in individuals from each family type, together with the mean number, are shown.

	POLE (n=7)	POLD1 (n=5)	MUTYH bi-allelic (n=2)	MUTYH mono-allelic (n=6)	Controls (n=12)
C:G>A:T	272	56	32	24	66
C:G>G:C	36	21	11	34	50
C:G>T:A	228	103	48	135	275
T:A>A:T	44	25	7	25	55
T:A>C:G	215	95	27	99	174
T:A>G:C	145	26	3	30	59
