# Supplementary material for: Germline de novo mutations in families with Mendelian cancer syndromes caused by defects in DNA repair
Source: Nat Commun. 2023 Jun 19;14:3636. doi: 10.1038/s41467-023-39248-0 (PMC10279637; doi:10.1038/s41467-023-39248-0)
Supplement: Supplementary file 3 — Reporting Summary [file 41467_2023_39248_MOESM3_ESM.pdf]

nature portfolio

Corresponding author(s): Ian Tomlinson

Last updated by author(s): Nov 28, 2022

Reporting Summary

Nature Portfolio wishes to improve the reproducibility of the work that we publish. This form provides structure for consistency and transparency in reporting. For further information on Nature Portfolio policies, see our Editorial Policies and the Editorial Policy Checklist.

Statistics

For all statistical analyses, confirm that the following items are present in the figure legend, table legend, main text, or Methods section.

n/a	Confirmed	
☐	☒	The exact sample size (*n*) for each experimental group/condition, given as a discrete number and unit of measurement
☒	☐	A statement on whether measurements were taken from distinct samples or whether the same sample was measured repeatedly
☐	☒	The statistical test(s) used AND whether they are one- or two-sided<br>*Only common tests should be described solely by name; describe more complex techniques in the Methods section.*
☐	☒	A description of all covariates tested
☐	☒	A description of any assumptions or corrections, such as tests of normality and adjustment for multiple comparisons
☐	☒	A full description of the statistical parameters including central tendency (e.g. means) or other basic estimates (e.g. regression coefficient) AND variation (e.g. standard deviation) or associated estimates of uncertainty (e.g. confidence intervals)
☐	☒	For null hypothesis testing, the test statistic (e.g. *F*, *t*, *r*) with confidence intervals, effect sizes, degrees of freedom and *P* value noted<br>*Give P values as exact values whenever suitable.*
☒	☐	For Bayesian analysis, information on the choice of priors and Markov chain Monte Carlo settings
☒	☐	For hierarchical and complex designs, identification of the appropriate level for tests and full reporting of outcomes
☐	☒	Estimates of effect sizes (e.g. Cohen's *d*, Pearson's *r*), indicating how they were calculated

Our web collection on statistics for biologists contains articles on many of the points above.

Software and code

Policy information about availability of computer code

Data collection	WGS using PCR-free library preparation at 30X coverage, with two independent libraries constructed per person from the same DNA sample. No failed experiments.
Data analysis	BWA (v 0.7.16)<br><br>CutAdapt (v 1.9.1)<br><br>Picard (v2.17.11)<br><br>Genome Analysis Toolkit (GATK) (v 4.0.10.1)<br><br>Samtools (v1.6)<br><br>DeNovoGear (v 1.1.1)<br><br>Ensembl Variant Effect Predictor (VEP) (v 97)<br><br>Integrative Genomics Viewer (IGV) browser (v 2.3.900)<br><br>SHAPEIT v2.r837

SigProfiler (v 1.1.3)

R (v 4.1.0)

For manuscripts utilizing custom algorithms or software that are central to the research but not yet described in published literature, software must be made available to editors and reviewers. We strongly encourage code deposition in a community repository (e.g. GitHub). See the Nature Portfolio guidelines for submitting code & software for further information.

Data

Policy information about availability of data

All manuscripts must include a data availability statement. This statement should provide the following information, where applicable:

- Accession codes, unique identifiers, or web links for publicly available datasets
- A description of any restrictions on data availability
- For clinical datasets or third party data, please ensure that the statement adheres to our policy

Access to all data will be provided, subject to formal agreements put in place to protect patient privacy and to fulfil ethical permissions. Access to case family data can be requested through access.crc.gwas.data@outlook.com. Access to Generations Scotland Data can be requested via access@generationscotland.org.

Human research participants

Policy information about studies involving human research participants and Sex and Gender in Research.

Reporting on sex and gender	Sex-specific analyses, in the form of maternal and paternal contributions to de novo mutation burden were an important part of the study. Other analyses were not reported with respect to sex or gender.
Population characteristics	All relevant population characeristics are described in the manuscript.
Recruitment	Recruitment to the CORGI study and Generations Scotland is described in references cited in the manuscript.
Ethics oversight	CORGI and CORGI2 were approved by South Central Hampshire A Research Ethics Research Ethics Committee and South Central Oxford A Research Committee, references 17/SC/0079, 06/Q1702/92 respectively. Ethical approval for the GS:SFHS study was obtained from the Tayside Committee on Medical Research Ethics (on behalf of the National Health Service), reference 05/S1401/89. All patients provided written, informed consent to taking part.

Note that full information on the approval of the study protocol must also be provided in the manuscript.

Field-specific reporting

Please select the one below that is the best fit for your research. If you are not sure, read the appropriate sections before making your selection.

☒ Life sciences ☐ Behavioural & social sciences ☐ Ecological, evolutionary & environmental sciences

For a reference copy of the document with all sections, see nature.com/documents/nr-reporting-summary-flat.pdf

Life sciences study design

All studies must disclose on these points even when the disclosure is negative.

Sample size	Sample size was determined by the available families recruited to the study, subject to this providing sufficient information to achieve the study goals.
Data exclusions	None
Replication	Use of duplicate DNA sequencing libraries and conformation of automated variant calling with visual inspection.
Randomization	Not applicable. This is a genetic case v control analysis.
Blinding	At the stage of visual inspection of the DNMs, one reviewer was blinded as to the identity of the person being sequenced for a subset of sequences. Any discrepancies were resolved by consensus. Since calling necessarily involves trios, blinding of parent/child status was not possible.

Reporting for specific materials, systems and methods

We require information from authors about some types of materials, experimental systems and methods used in many studies. Here, indicate whether each material, system or method listed is relevant to your study. If you are not sure if a list item applies to your research, read the appropriate section before selecting a response.

Materials & experimental systems

n/a	Involved in the study
☒	☐ Antibodies
☒	☐ Eukaryotic cell lines
☒	☐ Palaeontology and archaeology
☒	☐ Animals and other organisms
☒	☐ Clinical data
☒	☐ Dual use research of concern

Methods

n/a	Involved in the study
☒	☐ ChIP-seq
☒	☐ Flow cytometry
☒	☐ MRI-based neuroimaging
